# Leishmanicidal, cytotoxicity and wound healing potential of *Arrabidaea chica* Verlot

**DOI:** 10.1186/s12906-015-0973-0

**Published:** 2016-01-04

**Authors:** Joicy Cortez de Sá, Fernando Almeida-Souza, Renata Mondêgo-Oliveira, Iara dos Santos da Silva Oliveira, Lyah Lamarck, Isadora de Fátima Braga Magalhães, Aarão Filipe Ataídes-Lima, Higor da Silva Ferreira, Ana Lucia Abreu-Silva

**Affiliations:** 1 Rede Nordeste de Biotecnologia, Universidade Estadual do Maranhão, São Luís, Brazil; 2 Laboratório de Imunomodulação e Protozoologia, Fundação Oswaldo Cruz, Rio de Janeiro, Brazil; 3 Programa de Pós-Graduação Mestrado em Ciência Animal, Universidade Estadual do Maranhão, São Luís, Brazil; 4 Curso de Medicina Veterinária, Universidade Estadual do Maranhão, São Luís, Brazil; 5 Departamento de Patologia, Universidade Estadual do Maranhão, São Luís, Brazil

**Keywords:** *Arrabidaea chica*, Cytotoxicity, Healing, Leishmanicidal activity

## Abstract

**Background:**

Leishmaniasis includes a wide complex of diseases that affect humans and other mammals, and can range from a mild cutaneous form to a severe visceral type. The safety of the standard treatment using pentavalent antimony is a concern due to its toxic effects. The search for alternative, effective and less toxic treatments has led to the testing of natural products. The present study aimed to evaluate the cytotoxic, leishmanicidal and healing potential of *Arrabidaea chica*.

**Methods:**

The crude ethanolic extract, as well as the chloroform, methanol and ethyl acetate fractions of *A. chica* were prepared and phytochemical analysis was performed. Cytotoxic evaluation was carried out through MTT colorimetric assay, and the 50 % cellular cytotoxicity was determined. After that, the effect of the extract and fractions against Leishmania amazonensis promastigotes, at intervals of 24, 48 and 72 h, was analyzed, and 50 % inhibitory concentration was determined. The healing effect of the plant was also tested in surgical lesions in Swiss mice skin.

**Results:**

Phytochemical screening showed that the crude extracts contained flavonoids, tannins, anthocyanidins and chalcones. The leishmanicidal potential of *A. chica* produced satisfactory results in concentrations of between 60 and 155.9 μg/mL. Cytotoxic assay revealed a 50 % reduction in viable cells at a concentration of 189.9 μg/mL. The healing results indicated that the treated group exhibited more pronounced signs of lesion resolution in the early period, but this pattern did not persist throughout the treatment.

**Conclusions:**

The results of the present study demonstrate that *A. chica* has cytotoxic and leishmanicidal potential but its healing effect must be better studied.

## Background

Leishmaniasis is a group of diseases that affect several species of mammals that live in tropical and subtropical regions. Literature describes more than 20 species of the protozoan *Leishmania* that causes leishmaniasis, including cutaneous and visceral leishmaniasis [1, 2]. These clinical forms differ in clinical presentation, morbidity and mortality [3].

According to the World Health Organization, neglected diseases are those associated with low social indicators, and are thus more frequently found in developing countries where there is a lack of resources aimed at control and treatment and little research into finding an effective solution [4].

Among the clinical forms of the disease, the cutaneous, characterized by skin lesions, chiefly in exposed body parts, is the most common [5]. Depending on the species of *Leishmania*, in some patients leishmaniasis can heal without treatment, while in others diffuse cutaneous or mucocutaneous forms can develop [6].

In all clinical forms, pentavalent antimonials have been used as a mainstay therapy: the available drug for the treatment of leishmaniasis in the United States and Europe is sodium stibogluconate (Pentostam), while meglumine antimonate (Glucantime®) is more used in South America and Africa [7]. Despite this therapy having been widely employed since 1912, the mechanism of action is poorly understood and adverse effects, which include renal and hepatic toxicity, are severe [8]. Other limitations of this therapy are the necessity of daily parenteral administration and the resistance of the drug [2].

These limitations, the vast Brazilian biodiversity and traditional knowledge has led to the establishment of research networks to evaluate the leishmanicidal effect of plants from various biomes, such as the Cerrado and Amazon. It is therefore very important to perform experiments using natural products in order to identify new, less toxic, compounds which could be taken orally. In addition, the Brazilian government has created the RENISUS (Rede nacional de plantas medicinais de interesse do sistema único de saúde), a list of plants that can be used in the Unified Health Service, and *Arrabidaea chica* is included in this list.

*Arrabidaea chica* Verlot, popularly known as pariri, belongs to the Bignoniaceae Family, popularly known as *pariri*. It is endemic in almost all of Brazil but is most frequently found in the Amazon Forest [9] where it is used to treat skin disease, mulligrubs, anemia, jaundice and inflammatory reaction [10–12]. Widespread use by native populations lead researchers from different countries to evaluate the medicinal properties of this plant, including its anti-inflammatory [13]; astringent, antioxidant [14, 15]; anti-ulcer [16]; antimicrobial, antifungal [17, 18] and wound healing [19, 20] effects.

The aim of the present study was to evaluate the phytochemical effects, wound healing capacity and cytotoxicity of *Arrabidaea chica*, as well to evaluate the action of the crude extract and fractions of these plants on promastigotes of *L. amazonensis*.

## Methods

### Ethics committee

The experimental protocol for this study was approved by the ethics committee for animal research of the Universidade Estadual do Maranhão (CEUA-UEMA protocol 26/2009) and by the ethics committee in animal experimentation of Instituto Oswaldo Cruz - FIOCRUZ/RJ (CEUA-FIOCRUZ protocol LW72/12).

### Plant

Leaf and stem samples of *Arrabidaea chica* Verlot. were cultivated and collected in the Atico Seabra Herbarium, Departmento de Farmácia, Universidade Federal do Maranhão (São Luís, Maranhão, Brazil). The plant material was identified by Dr. Ana Zélia Silva and a voucher specimen was deposited under the number 1067. The plant material was collected between August and November 2011, which represents the dry season in this region, with a rainfall rate of 900 mm and an average temperature of 28 °C.

### Preparation of crude extract and fractions

To obtain the crude ethanolic extract, the samples were dried at 40 °C with forced air circulation. The dried material from each part of *A. chica* was macerated and immersed in ethyl alcohol, in a proportion of 3:1 (alcohol and plant material, respectively). In order to obtain the ethanolic extracts solvent removal was carried out by rotoevaporation, under reduced pressure at 30 to 40 °C, until the desired concentrations were achieved. The samples were stored under refrigeration and protected from light until analysis.

To determine the dry weight of the samples three aliquots of 0.5 mL of crude ethanolic extract were used. The yield of samples was calculated using the total weight of the powder, the dry weight obtained and the total final volume of the concentrated extract.

Additionally, the crude ethanolic extracts were fractionated by sequential partition using solvents of increasing polarity: chloroform, ethyl acetate and methanol. Extraction was carried out in the solid phase using silica gel, grail, pistil and approximately 500 mL of each solvent. Once obtained, the fractions were submitted to leishmanicidal and cytotoxic tests. Those that produced satisfactory results were sent to the Analytical Center of the Universidade de São Paulo (São Paulo, Brazil), to identify the bioactive compounds through gas chromatography and mass spectrometry.

### Phytochemical analysis

To evaluate the presence of secondary metabolites (saponins, anthocyanidins, chalcones, phenols, tannins, flavonoids, steroids, triterpenoids and anthraquinones), crude ethanolic extracts of *A. chica* were solubilized in methanol (in a concentration of 100 mg/5 mL), according to Costa and Matos [21, 22].

### *In vitro* cytotoxic assay

#### Obtaining of peritoneal macrophages and cell culture

Peritoneal macrophages were obtained from 4-week-old female BALB/c mice, from the Laboratory Animal Breeding Center (CECAL-FIOCRUZ) and kept in the Hélio and Peggy Pereira Pavilion animal facilitiy, located at the Oswaldo Cruz Foundation (FIOCRUZ, Rio de Janeiro, Brazil).

Three mL of 3 % sodium thioglycollate was inoculated into the peritoneal cavity of the animals and, after 72 h, were euthanized in a CO_2_ chamber. For macrophage recovery, 5 mL of a cold and sterile pH 7, 2 PBS solution was injected into the abdominal cavity, followed by massage and collection of lavage, which was kept in a falcon tube, placed in an icebox. After collection, the lavage was analyzed under an optical microscope to verify the presence of macrophages and the lack of red blood cells and bacteria.

Peritoneal lavage was centrifuged at 500 xg for 10 min at 4 °C. The supernatant was discarded and the cells resuspended in 1 mL of RPMI 1640 and counted in a Neubauer chamber. Final macrophage concentration was standardized at 5x10^5^ cells/well. For cell culture, RPMI supplemented with 10 % fetal bovine serum, L-glutamine (20 mM), sodium bicarbonate solution (7.5 %), penicillin (100 μg/mL) and streptomycin (50 μg/mL) was used, incubated at 37 °C with 5 % CO_2_.

#### CC_50_ determination

Cytotoxicity was measured by MTT colorimetric assay, in accordance with [23]. Briefly, 100 μL of peritoneal macrophages (5 × 10^5^ cells/well) were added to 96-well plates and incubated for 2 h, at 37 °C with 5 % CO_2_. After that, 100 μL of the crude ethanolic extracts or fractions of *A. chica* were added to the wells in serial concentrations (600 to 0.97 μL/mL). For each concentration a negative control was maintained. Dimethyl sulfoxide (DMSO) was used as a control drug in serial dilutions starting at 20 %. After 24 h of incubation, 5 μL of MTT [3-(4, 5-dimethylthiazol-2-yl)-2, 5-diphenyl tetrazolium bromide] was added to each well, then the plates were incubated again for 2–4 h under the same conditions. Later, they were centrifuged at 1500 xg and 200 μL of each well supernatant was discarded, before 100 μL of DMSO was added. Absorbance was measured in a spectrophotometer, at a wavelength of 540 nm. 50 % cellular cytotoxicity (CC_50_) was calculated using GraphPad Prism 5.

### *In vitro* antileishmanial activity

#### Parasites

The present study used the *Leishmania amazonensis* (MHOM/BR/76/MA-76 – IOC/FIOCRUZ-RJ) strain, isolated from a patient with diffuse cutaneous leishmaniasis and maintained by serial passages in BALB/c mice, and periodically re-isolated in culture medium. Promastigote forms were grown at 26 °C in Schneider’s medium, supplemented with 10 % fetal bovine serum, streptomycin (100 μg/mL) and penicillin (100 μg/mL). To ensure infectivity only cultures of promastigote with a maximum of seven *in vitro* passages were used.

Before evaluation of leishmanicidal activity and determination of the 50 % inhibitory concentration (IC_50_) of *A. chica*, a screening test was performed to determine the concentration used in IC_50_ assay. The concentration used ranged between 500 and 0.97 μg of crude ethanolic extracts or fractions, diluted in 1 % DMSO, and added to 96-well plates containing RPMI, serially and in triplicate. Wells containing medium and parasites, medium only, and the reference drug, Amphotericin B (2 μg/mL), were used as controls. 100 μL of *L. amazonensis* promastigotes in the logarithmic phase (1x10^6^ parasites/mL) were added to each well, and the plate was incubated at 26 °C. Parasite viability was measured after 24, 48 and 72 h, by observing motility under an inverted microscope. The parasites were classified according to mobility on a scale of zero to ten, where zero means no viable forms and ten, viable forms.

Based on the screening results the IC_50_ was determined, following the same aforementioned protocol, and leishmanicidal activity was assessed by counting the viable parasites in a Neubauer chamber. The data was then normalized using the formula: Percentage of growth inhibition = number of parasites in test wells/number of parasites in the control well, multiplied by 100; and used for statistical analysis.

### Wound healing activity

#### Cream and therapeutic protocol

Two topical preparations (10 mg/g and 20 mg/g) were prepared containing the crude ethanolic extract from *A. chica* leaves mixed with Lanette® cream, as a water-soluble anionic vehicle.

Forty 4-to-6 week old male Swiss Webster mice from the animal facility of the Universidade Estadual do Maranhão (São Luís, Maranhão, Brazil) were used in this experiment. The animals were randomly divided into two groups (*n* = 20 each). The control group was treated with Lanette® cream and the experimental group was treated with Lanette® cream containing the crude ethanolic extract of *A. chica*.

For creation of a dorsal skin wound, the animals were anesthetized with an intraperitoneal injection of xylazine hydrochloride (10 to 15 mg/kg) and ketamine hydrochloride (100 to 150 mg/kg). Trichotomy of the dorsal region and demarcation of the skin with a punch was then carried out. Resection of a circular segment of approximately 6 mm diameter was performed, exposing the dorsal fascia. Hemostasis was carried out by digital compression with sterile gauze, with treatment initiated immediately afterwards.

The therapeutic protocol was performed twice a day throughout the experimental period. Transverse and longitudinal diameters of the lesions were measured daily, using a digital caliper, and monitored through photography. Skin wound areas were calculated using the formula A = π x R x r, in which A represents the area, π is equal to 3.14, R represents the largest diameter and r the smallest diameter of the wound.

#### Histological analysis

After 3, 7, 14 and 21 days of treatment, the mice were euthanized and the lesions were excised with a margin of 1 cm of integrate skin, stored in 10 % buffered formalin solution and submitted to histological procedures. After embedding in paraffin, 5 μm thick cross sections were cut and stained with hematoxylin-eosin (HE) for morphological analysis of the tissue. Additionally, fragments of liver, spleen and kidney were collected, for evaluation of potential cytotoxic lesions.

Slides were analyzed by light microscopy by two independent examiners, following the parameters: crust formation, presence of inflammatory infiltrate, angiogenesis, reepithelialization and collagen formation [24]. These features were graded as absent, mild, moderate and severe, according to their appearance.

#### Statistical analysis

Statistical analysis was performed using GraphPad Prism 5.0.4 (GraphPad Software Inc.). IC_50_ and CC_50_ values were calculated from the mean percentage reduction of promastigotes and macrophages, respectively. Curves were determined by application of sigmoidal regression to logarithm concentration response data. *In vitro* results are representative of three different experiments, performed in triplicate, under the same temperature, CO_2_, time and culture medium conditions, with similar results. Analysis of wound healing was performed using the Student’s t-test. Histological variables were analyzed by non-parametric test. Differences were considered significant at *P* < 0.05.

## Results

### Yield of extraction and phytochemical analysis

The crude ethanolic extracts of *Arrabidaea chica* leaves and stem presented a yield of 59.2 and 3.33 % respectively, which shows that the stem has a low extraction efficiency, as the same extraction method was used for both stem and leaves.

Phytochemical screening showed that both leaves and stem extracts contain flavonoids, tannins, anthocyanidins and chalcones. Phenolic compounds were found only in the leaves (Table 1).

**Table 1:** Phytochemical prospection of crude ethanolic extract of leaves and stem of *Arrabidaea chica*, collected in Maranhão state, Brazil

Metabolite class	Reactions	*Arrabidaea chica*	
		Leaves	Stem
Anthraquinones	NaOH	-	-
Flavonoids	AlCl_3_	+	+
Triterpenes/steroids	Anhidrous acetic/sulphuric acid	-	-
Saponin	Foaming index	-	-
Phenolic compound	FeCl_3_	+	-
Tannin	Gelatin	+	+
Anthocyanidins/chalcones	HCl/NaOH	+	+

(+) presence; (−) absence

### IC_50_ determination

In screening tests to evaluate the potential leishmanicidal effect on *L. amazonensis* promastigotes, *A. chica* stem extract showed no activity against the protozoan, even at the highest tested concentration (500 mg/mL), and so was not used in continuity tests.

**Fig. 1:**
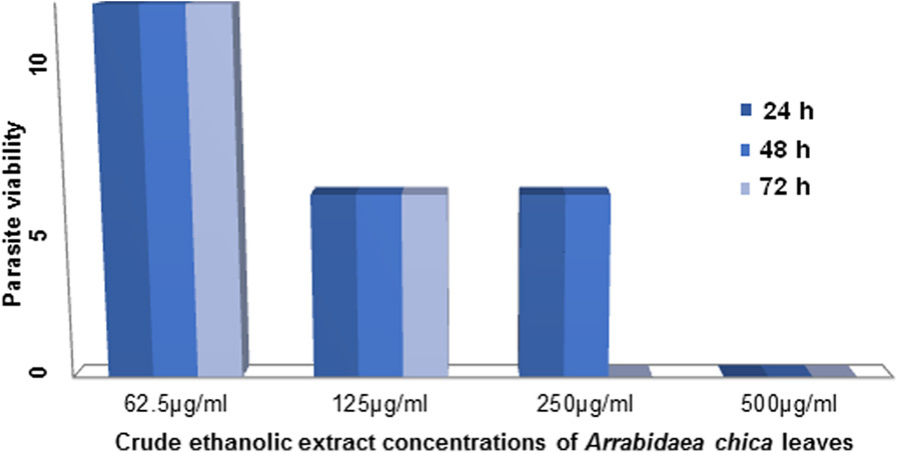
Viability of *Leishmania amazonensis* promastigotes cultivated in RPMI media at 24 °C and treated with crude extract of leaves of *Arrabidaea chica,* at concentrations of 62.5 to 500 μg/mL. Score: (10) viable, (5) less viable, (0) unfeasible

In the screening tests of the crude ethanolic extract *of A. chica* leaves, there was a reduction of 50 % of *L. amazonensis* promastigote viability with a concentration of 125 μg/mL (Fig. 1) at the intervals of 24, 48 and 72 h after incubation. However, the concentration of 250 μg/mL displayed antileishmanial activity in the first 24 h of treatment and a total absence of viable parasites after 72 h of incubation. The concentration of 500 μg/mL of the leaf ethanol extract had the best leishmanicidal efficacy at the three tested intervals.

**Fig. 2:**
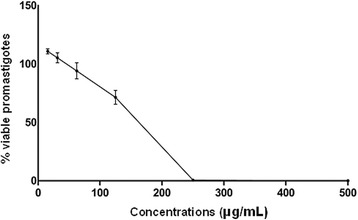
*In vitro* leishmanicidal activity of the crude ethanolic extract of *Arrabidaea chica* against *Leishmania amazonensis* promastigotes at concentrations of 0.97 to 500 μg/mL

After screening, the 50 % inhibitory concentration (IC_50_) of *L. amazonensis* promastigote forms was determined as 155.9 μg/mL (Fig. 2).

When evaluating the leishmanicidal activity of the chloroform, methanol and ethyl acetate fractions, there was a 50 % reduction of *L. amazonensis* promastigotes at concentrations of 120 μg/mL, 120 μg/mL and 60 μg/mL, respectively (Table 2). This result demonstrated that the fractions acted against the parasite at lower concentrations than the crude extract, and that the ethyl acetate fraction had the most effective result.

**Table 2:** 50 % inhibitory concentration (IC_50_) of *Leishmania amazonensis* promastigote forms after 72 h treatment with crude ethanolic extract and chloroform, methanolic and ethyl acetate fractions of *Arrabidaea chica*

Treatments	IC_50_ (μg/mL)
Crude ethanolic fraction	155.9 ± 0.1185
Chloroform fraction	>120
Methanolic fraction	>120
Ethyl acetate fraction	>60
Amphotericin B (reference drug)	0.76 ± 0.9218

Values represent mean ± standard deviation

### CC_50_ determination

**Fig. 3:**
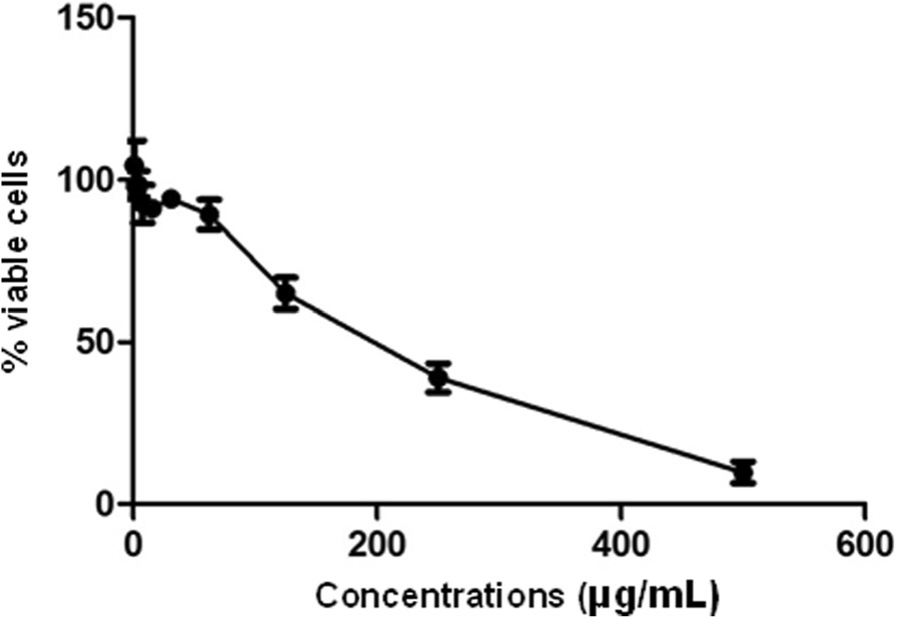
Cytotoxicity evaluation in peritoneal macrophages cultured in RPMI medium, at 37 °C and 5 % CO2 and treated for 24 h with ethanol extract of *Arrabidaea chica* leaves at concentrations of 0.97 to 500 μg/mL

Cytotoxicity assay in the peritoneal macrophages after 24 h showed that a 189.9 μg/mL concentration of *A. chica* leaf extract reduced the number of viable cells by 50 %, representing CC_50_ (Fig. 3).

Regarding CC_50_ and IC_50_ values of the crude extract, it was observed that *A. chica*’s bioactive compounds have a higher Selectivity Index (1.218) to the parasite than to the host cell, proving to be a potential treatment for leishmaniasis.

### Healing effect

Regarding the evaluation of dermal irritation potential of the crude extract of *A. chica* leaves, a concentration of 20 mg/g caused skin irritation, with the presence of moderate and persistent erythema during the treatment period. Thus, the healing test was performed with a concentration of 10 mg/g.

**Fig. 4:**
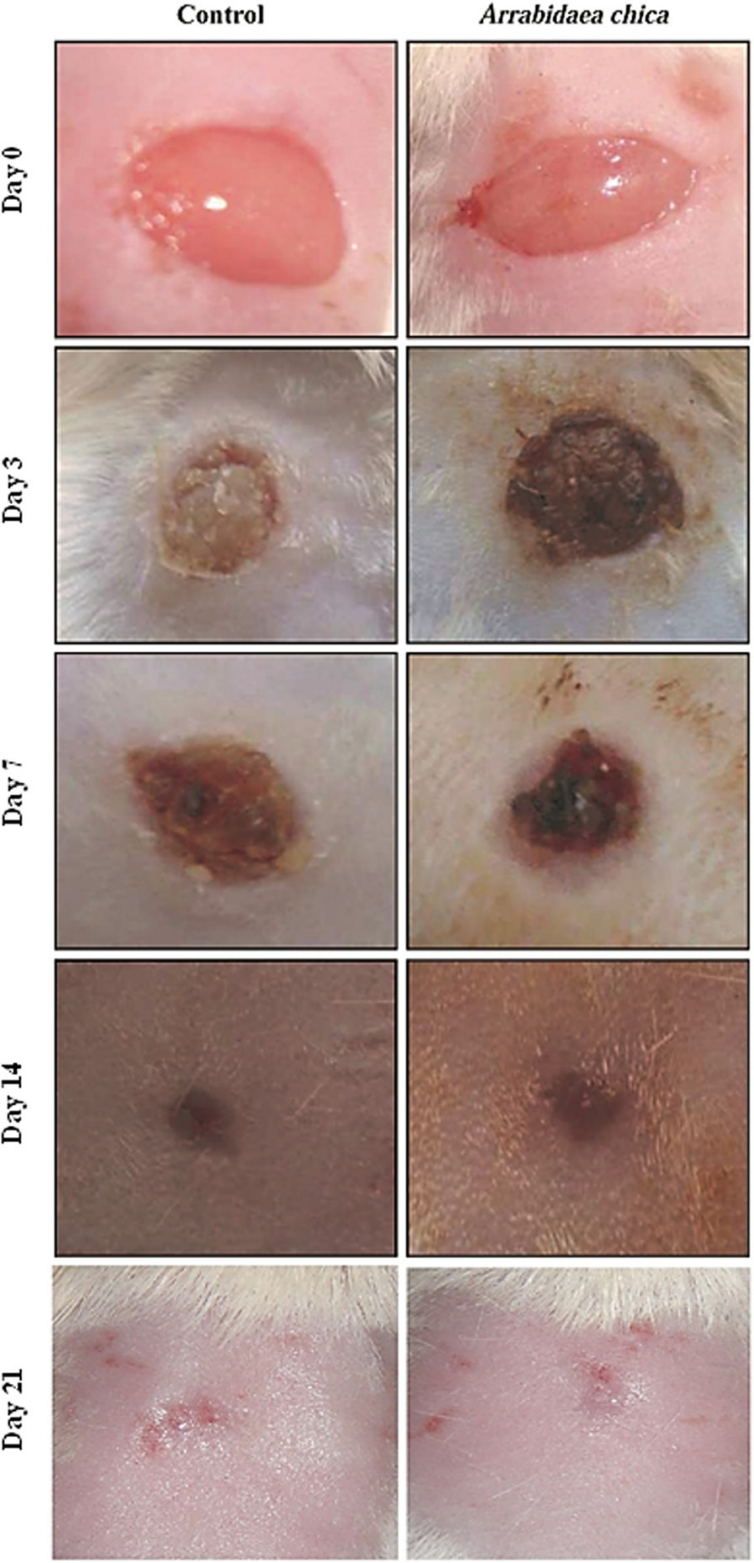
Macroscopic evaluation of the healing process in Swiss mice Webster treated with LANETTE® cream (Control Group) and ethanol extract of *Arrabidaea chica* leaves (Experimental Group) over 21 days

In macroscopic analysis of the wound healing process, the beginning of the tissue repair process, with the presence of crusts on the third day, was noted. On the seventh day, a reduction in the size of the wound and the formation of granulation tissue in both the treated and control groups was observed (Fig. 4).

**Fig. 5:**
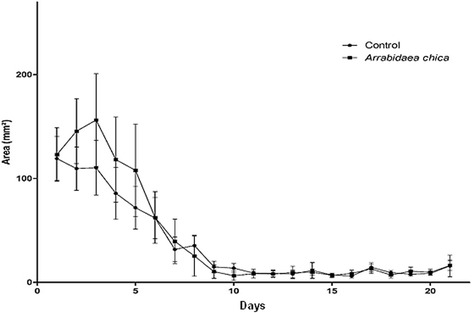
Wound healing kinetics of surgical lesions in Swiss Webster mice treated with Lanette® cream (Control Group) and ethanol extract of *Arrabidaea chica* leaves (Experimental Group) over 21 days

In the 0–3 day interval an increase in the lesion area was observed in the group treated with *A. chica,* as compared to the control group (Fig. 5). In the third to the 10th day, there was a marked reduction in the wound area, with a gradual approximation of the edges in both the control and treated groups.

Between the 14th and 21th post-injury days, the retraction of the wound was almost complete, with marked healing and tissue repair.

**Fig. 6:**
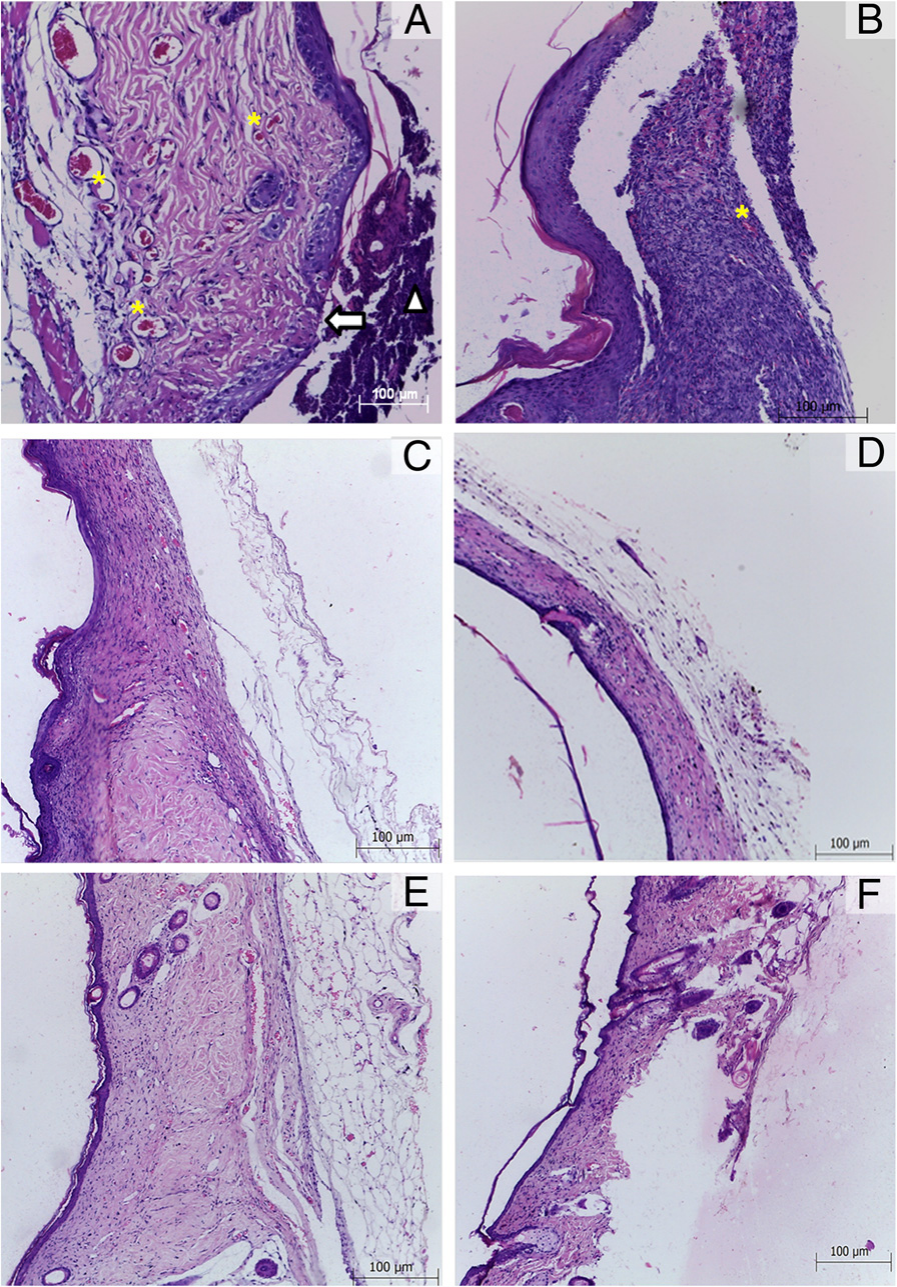
Swiss Webster. Skin. Healing process through topical treatment with a cream containing the crude ethanolic extract of *A. chica* (experimental group) or only Lanette® cream (control group). **a** (3 days) - Experimental group: cellular debris (Δ), reepithelization (arrow), angiogenesis (*). **b** (7 days) - Experimental group: Absence of cellular debris and angiogenesis (*). **c** (14 days) and (**d**) (21 days) - Control group: Absence of cellular debris and reepithelization. **e** (14 days) and (**f**) (21 days) - Experimental group: Absence of cellular debris and reepithelization. HE

On the third day of treatment cellular debris and moderate to intense inflammatory process was observed in both groups. Discrete angiogenesis, collagen deposition and epithelialization were observed in the treated group (Fig. 6a–b), as well as an increased amount of mast cells.

On the seventh day of treatment, re-epithelialization and the reduction of cell debris and the inflammatory process was quite evident. While there was no difference between the two groups, the patterns of re-epithelialization, angiogenesis and collagen deposition were higher in the control group.

Healing process evaluation between the 14th and 21st days showed epithelialization and intense collagen deposition in the control group (Fig. 6c–d), while the treated group (Fig. 6e–f) revealed patterns ranging from mild to intense. In both groups, there was a low number of inflammatory cells and mast cells. Angiogenesis was discreet in the treated group and varied from moderate to mild between the 14th and the 21st day.

The topical preparation with the crude extract of *A. chica* at a concentration of 10 mg/g displayed a more effective healing activity in the initial phase of the experiment, but was not able to induce a stronger response throughout the 21 day observation period.

## Discussion

Plants are important sources of bioactive compounds, and are a major potential source of new therapeutic agents against several diseases, including those of interest to public health, in particular neglected diseases such as leishmaniasis.

The bioactive compounds produced by plant cells may vary depending on soil, climate and the season that the plant, fruit or leaves were collected. Soil and climate conditions, along with the plant’s need for adaptation and defense, bring about this change in chemical constituents [25]. Phytochemical analysis of *A. chica* revealed the presence of flavonoids, tannins, anthocyanidins, chalcones and phenolic compounds, as previously described for this species [26, 27].

The first chemical study with the leaves of *A. chica* was performed by Chapman et al. and reported the isolation of the substance 3-deoxyanthocyanidin, more specifically 1,6,7-dihydroxy-5,4′-dimethoxy-flavylium (carajurin) and 6,7,4′-trihydroxy-5′, methoxy-flavyluim (carajurone), responsible for the reddish color of the extract [28]. In other studies, other compounds, such as flavone acacetin, oleanolic triterpene acid and two deoxiantocyanidins (6, 7, 3′-4′-tetrahydroxy-5-methoxy-flavylium and 6, 7, 3′-trihydroxy-5, 4′ dimethoxy-flavyliume) were isolated [29-31].

Anthocyanins are plant pigments belonging to the family of flavonoids, included in the group of phenolic compounds [32, 33]. More than 50 new anthocyanins have been isolated from the flowers, fruits, seeds and leaves of plants. They play a pivotal role in flower pollination, seed dispersal by insects and defense against predatory insects [34].

Pharmacological studies with anthocyanins reveal their high antioxidant effect [15] on metabolic diseases [35]. The antioxidant, cytotoxic, antimicrobial and diuretic potential of the ethanol extract and fractions of *A. chica* was evaluated by Amaral et al. that confirmed this antioxidant and diuretic action, and the absence of an antimicrobial and cytotoxic response [14]. The therapeutic activity of the phenolic compounds is mainly attributed to their antioxidant capacity, the chelation of metal ions, modulation of gene expression and interaction with cell signaling pathways [36].

Phenolic composition and antioxidant activity of the leaves of *A. chica* was evaluated by Siraichi et al. by liquid chromatography-electrospray ionization-tandem mass spectrometry (LC-ESI-MS/MS), and verified the presence of two important flavonoids, apigenin and scutellarein, which are most responsible for the significant antioxidant action [15].

In pharmacological tests, Alves et al. determined the chemical potential of *A. chica* dye, which is widely used by indigenous tribes, and verified the presence of alkaloids, anthocyanidins, anthocyanins, anthraquinone, steroids, triterpenoids, phenols, flavanonois, flavanols, flavanones, saponins and catechin tannin [37]. This study described some compounds undetected in the present study, differing in the presence of anthraquinones, steroids, triterpenoids and saponins. This fact is probably due to the climate differences between the two places where the plant was collected, the states of Maranhão and Pará, as well as the method by which the extract was obtained. Both were obtained using ethanol as a solvent; however, in the present study this was submitted to 72 h rotoevaporation at 40 °C, while in the aforementioned study the extract was macerated for 10 days in a capped percolator.

In vitro pharmacological studies have demonstrated the antineoplasic and antioxidant potential of *A. chica*, and this action was directly related to the high content of flavonoids, especially anthocyanins [38]. These and other benefits made the search for new sources of anthocyanins essential [35].

The secondary metabolites in *A. chica*, such as flavonoids, the antimicrobial, antiviral, anti-inflammatory, antineoplastic, antifungal and anti-protozoan action of which has already been reported in literature [17, 38–40], encourages many researchers to study this plant.

In the present study, the leishmanicidal potential of the crude extract and fractions of *A. chica*, which displayed satisfactory results at concentrations between 60 and 155.9 μg/ml, depending on the type of fraction. The chemical constituents of the plant were more selective for the protozoan than the host cell, with a cytotoxicity effect at concentrations higher than those with leishmanicidal action.

Several studies have aimed to identify and characterize crude extracts, fractions and essential oils [41-48], as well as the isolated compounds of the plant [49, 50], with leishmanicidal activity. The screening of these natural compounds is performed with cultures of *Leishmania* spp., as it is easy to maintain and produce [51].

According to the World Health Organization, a candidate drug must undergo *in vitro* cytotoxicity and antiparasitic activity testing. After this initial screening, the results are evaluated to define the continuity of studies and the possibility of *in vivo* assays [52].

Studies performed by Rodrigues et al. [53] investigated the leishmanicidal effect of five fractions obtained from the crude hexane extract of *A. chica*, on *L. amazonensis* and *L. infantum* promastigote forms, by determining the minimum inhibitory concentration (MIC), which was found to be 37.2 and 18.6 μg/ml, respectively. This result is related to one of the fractions used, the main chemical components of which were linoleic acid methyl ester (25.38 %), n-hexadecanoic acid (19.61 %), octadecanoic acid (14.10 %) and gamma-sitosterol (12.85 %). The study also evaluated ultrastructural alterations of the parasite under the influence of this fraction, and observed mitochondrial swelling, disruption of mitochondrial membrane, presence of vesicles in the cytoplasm, mitochondria and flagellar pocket and modifications in the Golgi complex.

The significant increase in studies of plant compounds with leishmanicidal potential is due to of the major importance of this disease, of which there are large numbers of new cases annually. According to the World Health Organization 1.3 million new cases are recorded per year, being 300,000 cases of visceral leishmaniasis and 1 million cutaneous leishmaniasis [5].

Leishmaniasis are characterized as neglected diseases, as they are associated with low social indicators and are more prevalent in developing countries, whose resources in the areas of control, treatment and research are insufficient [4].

Additionally, plant extracts or plant compounds can represent a valuable source of new medicinal agents and alternative, effective and less toxic treatments, compared to conventional drugs (pentavalent antimoniates, amphotericin B, pentamidine), leading to a search for natural products with a potential leishmanicidal action. Furthermore, the benefits obtained from the search for natural products have encouraged interest in valuable synthetic compounds [42].

The crude extract from the leaves of *A. chica* had a cytotoxic effect at a concentration of 189.9 μg/mL. Maliofete et al. [54], also testing the ethanol extract from the leaves of *A. chica*, observed low oral toxicity, absence of cytotoxicity and antibacterial activity against *Helicobacter pylori. In vivo* experimental studies did not show any clinical or histopathological signs of extract toxicity in the pleura [13], open or sutured wounds or burns [55]. Studies by Barbosa et al., assessing the antifungal, antimicrobial and antiprotozoal potential of the ethanolic extract and fractions of *A. chica*, observed growth inhibition of *Trichophyton mentagrophytes* and a significant trypanocidal effect against *Trypanosoma cruzi*, as well as the absence of relevant acute toxicity, even at the highest dose tested (1000 mg/kg) [17].

Several factors must be taken into consideration when evaluating the toxic effects of a plant extract, such as seasonal and environmental factors, the genotypic variations of the species, the part of the plant used to produce the extract, the time of collection and the age of the plant [25].

In traditional medicine in the Amazon region, *A. chica* is widely used as an anti-inflammatory, healing and anti-anemic medicine, and is prepared from the leaves as a tea, for oral or vaginal administration [16, 17, 56]. However, despite its wide popular use, several studies are still necessary to evaluate the medical potential of this plant.

In the present study, the healing potential of the crude extract of the leaves of *A. chica* in skin lesions was evaluated. A satisfactory response was initially observed, with angiogenesis and collagen deposition, but over the 21-day observation period it was found that the extract did not accelerate healing, presenting a similar profile to the control group.

Several local and systemic factors can cause variations in the healing process, leading to a delay or even prolongation of healing. Among these are the local of the lesion, existence of infection, surgical technique, tissue ischemia and bandages, as well as malnutrition and/or deficiency of trace minerals and vitamins [57, 58].

As cited above the importance of bandages in the healing process should be noted. Several studies have demonstrated that there the healing process is more successful when bandages are placed on the location of the wound after the application of the test product [16, 59] to protect the injury area. This step, which was not performed in the present study, may have contributed to the retardation of healing. The application of a suitable compress enables an increase of the natural debridement and simplifies healing, as it keeps the exudates rich on mediators on the wound surface [60].

Wound healing is a complex and dynamic process of the replacement of a devitalized tissue by a new tissue, which occurs through a series of biochemical cascade events; usually divided into three phases: the inflammatory, proliferative, and remodeling phases [61]. Wound healing treatment has improved considerably over time, notably in order to achieve the best scarring results in the shortest possible time.

In the present study, a strong inflammatory response was observed at the beginning of the healing process, with an increased lesion area until the 3rd day after surgical incision and the presence of inflammatory cells in histological evaluation. The inflammatory phase is characterized by increased vascular permeability and immune cells chemotaxis to the site of injury, due to the release of mediators (histamine, serotonin, bradykinin, prostaglandins, thromboxanes) [62].

The most pronounced reduction period in the wound area was between the third and the 10th day, with retraction of the wound area, representing the proliferative phase. During this phase fibroplasia, granulation tissue formation, and removal of cellular debris were observed, with the formation of a temporary repair tissue during inflammation, and development of tissue substitutes [63].

In the healing process, fibroplasia occurs together with neovascularization [61]. As the wound healing process advances, fibroblasts undergo phenotypic changes, with an abundance of rough endoplasmic reticulum, due to intense protein synthesis; and subsequent transformation into myofibroblasts, which help in the retraction of the lesion [64, 65].

Studies performed by Jorge et al. [16], when evaluating the healing, anti-inflammatory, antiulcerogenic and antioxidant effect of crude methanolic extract of *A. chica*, found that there was no reduction of paw edema induced by carrageenan or ear edema induced by croton oil in rats. However, *in vitro* fibroblast proliferation induction, stimulation of collagen synthesis *in vivo* and *in vitro*, anti-ulcer activity and moderate antioxidant capacity were observed.

After 10 days, it was verified that 96 % of wounds in the experimental group had healed, with a prominent collagen deposition, while in the control group, treated with saline solution, only 36 % of the wounds had healed, with a mild collagen deposition [16].

The topic healing effect of *A. chica* extract in the tendons of Wistar rats was investigated by Aro et al., that found better collagen organization in the treated groups than in the control group, as well as an increase in sulfate, on the 14th day treatment [20].

Compounds exhibiting antioxidant activity can indicate good therapeutic agents in the healing process, according to Houghton et al. [57]. Non-enzymatic antioxidant activity is promoted by flavonoids, carotenoids, ascorbic acid, glutathione, and vitamin E, among others [58, 66, 67]. Therefore, the healing activity of *A.chica* is attributed to the antioxidant activity of anthocyanins and to fibroblast proliferation stimuli and consequent collagen synthesis [16, 68].

Fibroblasts are connective tissue cells, essential for dermis formation, due to collagen production, and responsible for structural firmness. After tissue injury, fibroblasts near the lesion site proliferate, migrate to the wound and produce a large amount of matrix material rich in collagen (type I and III), which helps to isolate and repair the injured tissue [63].

The proliferation of fibroblasts is directly related to the presence of macrophages in the healing process, as these release growth factors and cytokines essential for the maturation of the inflammatory phase and the beginning of the healing. Among the growth factors responsible for the proliferation, insulin-like growth factor 1 (IGF-I) and transforming growth factor beta (TGF-β) play an important role. Studies have demonstrated that IGF-I increases the procollagen chain expression in cultured dermal fibroblasts [63].

In the present study, on day 14 the control and experimental groups presented a smaller healing area than on day 07. At the end of the experiment, after 21 days, both groups were in the final stages of the healing process, in which the remodeling phase predominates, characterized by the reduction of inflammatory cells [69] and blood vessels at the lesion site [61], being the stage in which the scar acquires maximum resistance [70].

Several plants are used in popular medicine for the treatment of skin disorders. A number of these, such as *Aloe vera* (“babosa”), *Schinus terebenthifolius* (“aroeira”), *Stryphnodendrom barbatiman* (“barbatimão”), *Calendula officinalis* (“calendula”) and *Triticum vulgare* (“trigo”) are especially worthy of note due to the number of Brazilian studies involving the phytopharmaceutical use of these species [71–74].

## Conclusions

The present study demonstrates that the compounds extracted from *A. chica* leaves are potential leishmanicidal agents in non-cytotoxic concentrations, with further research required to continue the evaluation of this potential, in order to develop new drugs based on these compounds, or associate them with drugs already used in therapeutic protocols for the disease. The wound healing potential of the plant requires additional studies, in order to eliminate the interference of external factors and optimize the action of *A. chica* extract.

## Abbreviations

°C: Celsius degree
μg/mL: microgram per milliliter
μL: microliter
μm: micrometer
μM: micromolar
AlCl_3_: Aluminum chloride
ANOVA: Analysis of variance
CC_50_: 50 % cellular cytotoxicity
CO_2_: Carbon dioxide
DMSO: Dimethyl sulfoxide
FeCl_3_: Iron chloride
FGF2: Fibroblast growth factor 2
G: giros
HCl: Hydrochloric acid
HE: Hematoxylin-eosin
IC_50_: 50 % inhibitory concentration
IGF-I: Insulin-like growth factor 1
mg/kg: milligrams per kilogram
mg/mL: milligrams per milliliter
mM: millimolar
MTT: (3-[4,5- dimethylthiazol-2-yl]-2,5-diphenyl tetrazolium bromide)
NaOH: Sodium hydroxide
PBS: Phosphate buffered saline
RPMI: Roswell Park Memorial Institute medium
TGF-β: Transforming growth factor beta
